# Impact of Anti-T-lymphocyte globulin dosing on GVHD and Immune reconstitution in matched unrelated myeloablative peripheral blood stem cell transplantation

**DOI:** 10.1038/s41409-022-01666-x

**Published:** 2022-07-13

**Authors:** Radwan Massoud, Evgeny Klyuchnikov, Nico Gagelmann, Tatiana Zabelina, Christine Wolschke, Francis Ayuk, Ulrike Fritzsche-Friedland, Axel Zander, Nicolaus Kröger

**Affiliations:** Department of Stem Cell Transplantation, University Medical Center Hamburg-Eppendorf, Hamburg, Germany

**Keywords:** Haematopoietic stem cells, Bone marrow transplantation, Allotransplantation

## Abstract

Data on the influence of different Anti-lymphocyte globulin (ATLG) doses on graft versus host disease (GVHD) incidence and immune reconstitution in matched unrelated (MUD) allogeneic Stem cell transplantation (allo-SCT) is limited. This retrospective study conducted at the University Medical-Center Hamburg compares GVHD and Immune reconstitution after myeloablative MUD (HLA 10/10) PBSC allogeneic stem cell transplant between 30 mg/Kg (*n* = 73) and 60 mg/Kg (*n* = 216) ATLG. Detailed phenotypes of T, B natural killer (NK), natural killer T (NKT) cells were analyzed by multicolor flow at day 30, 100, and 180 posttransplant. Neutrophil and platelet engraftments were significantly delayed in the 60 mg/kg group with a higher Cumulative incidence of Infections (67% vs 75% *p* = 0.049) and EBV (21% vs 41% *p* = 0.049) reactivation at day 100 in this group. In the 30 mg/kg group, we observed a faster reconstitution of naïve-B cells (*p* < 0.0001) and γδ T cells (*p* = 0.045) at day+30 and a faster naïve helper T-cell (*p* = 0.046), NK-cells (*p* = 0.035), and naïve B-cell reconstitution (*p* = 0.009) at day+180. There were no significant differences in aGVHD, cGVHD, NRM, RI, PFS, and OS between the groups. The choice of ATLG dose has significant impact on IR but not on GVHD after MUD-allo-SCT. Higher doses are associated with delayed engraftment and increased infections.

## Introduction

Due to the graft versus tumor effect, allogeneic stem cell transplantation (allo-SCT) is a potentially curative treatment strategy for hematological diseases [1, 2]. However its benefits may be offset by increased non related mortality (NRM), mainly due graft-versus-host-disease (GVHD) and infections [3]. The use of an unrelated donor and peripheral blood stem cells (PBSC) is associated with an increased risk of GVHD. Randomized studies have shown that pretransplant anti-T-Lymphocyte globulin (ATLG) can prevent severe acute and chronic GVHD [4–6]. Although lower ATLG doses may compromise the immunosuppression effects, higher ATLG doses may offset its benefit by decreasing antiviral and graft-versus-malignancy effect by depletion of donor effector T cells [7]. Even though the use of ATLG in allo-SCT is well established, however, data on optimal ATLG dosing in the setting of MUD-PBSC is scarce, only one study has compared different ATLG doses in this setting [8]. In the present study, we aim to compare the IR kinetics and transplant outcomes between recipients of 30 mg/Kg vs 60 mg/Kg ATLG as TCD strategies undergoing MUD-PBSC-allo-SCT with myeloablative conditioning (MAC).

## Materials and methods

This retrospective study conducted at University Medical Center Hamburg-Eppendorf (UKE) with a primary goal to compare IR between 30 mg/Kg (ATLG-30) vs. 60 mg/kg (ATLG-60) ATLG in recipients of MAC PBSC allo-SCT. The choice of ATLG dose was according to treating physician preference. Secondary outcomes included incidence of viral reactivations, engraftment, rate of infections and Infection related mortality (IRM), aGVHD, cGVHD, non relapse mortality (NRM), progression free survival (PFS), and overall survival (OS). All patients signed written informed consents for treatment and the study was approved by the institutional review board at UKE. To have comparable groups, we selected only patients receiving MAC with PBSC as stem cell source, and MUD (HLA 10/10).

MAC regimens were defined according to published working group definition [9]. ATLG (Grafalon®, Neovii, Switzerland) was given at a dose of 30 mg/kg or 60 mg/kg. A test dose of 200 mg was given at day −4 and the remaining ATLG doses were fractionated between days −3 to −1. Similar supportive care was used for all patients per institutional guidelines including antimicrobial prophylaxis consisting of fluoroquinolone for bacterial infections, trimethoprim-sulfamethoxazole or pentamidine for *Pnemocystis jiroveci*, micafungin for fungal infections and acyclovir for viral infections. Patients were screened weekly for CMV and EBV by blood PCR.

Neutrophil engraftment was defined as the first 3 consecutive days with a measure of absolute neutrophil count >0.5 × 10^9^/L. Platelet engraftment was defined as the first consecutive days with a platelet count >20 × 10^9^/L without transfusion support. Acute GVHD was graded according to standard criteria [10]. Chronic GVHD was graded according to National Institute of Health (NIH) criteria routinely at every visit after transplantation [11]. Infections were defined as any microbial testing with a positive result and requiring therapy at any time-point after allo-SCT.

As per institution guidelines, blood samples were collected for each patient on days +30, +100, and +180 post-allo-SCT. Routine analyses for absolute concentrations of CD3+, CD4+, CD8+, NK, and γδ T cells were performed by flow cytometry according to an internal protocol: (1) CD4-APC, CD8-PE, Multitest (CD3 FITC, CD16 + 56 PE, CD45 PerCP, CD19 APC); (2) CD4-APC, CD45-V450, Multitest (CD45RA FITC, CD45RO PE, CD3 PerCP, CD8 APC); (3) CD45-V450, CD3-PerCPl, anti-TCR-PE, anti-HLA DR-APC in peripheral blood samples. All antibodies were obtained from Becton Dickinson (BD Biosciences, New Jersey, USA). Up to 5000 events (25,000 per sample) were acquired per tube. Sample acquisition was performed using a BDTM FACS-Canto flow cytometer with the BDTM FACSDiva software which was also used for data analyses.Immunophenotypes were assessed using four color cytometry using mouse anti-human antibodies for the following cells: T-lymphocytes (CD3+), activated-T-lymphocytes (CD3+ HLADR+), T-helper (CD3+/CD4+), T-cytotoxic (CD3+/CD8+), B-lymphocytes (CD19+), B-lymphocytes subpopulations (CD19+CD5+ CD1d+)(CD19+ CD27+), naïve-B-cells (CD19+ CD27-CD10+), NK-cells (CD56+ CD3-), NKT-cells (CD56+ CD3+), naïve-T-helper (CD4+ CD45RA+), memory-T-helper (CD4+ CD45R0+), naïve-T-cytotoxic (CD8+ CD45RA+), memory-T-cytotoxic (CD8+ CD45R0+ ), γδT-cells (γδTCR+, CD3+), regulatory-T-cells (CD4+ CD25+ CD127low-neg).

### Statistical methods

All data was retrospectively collected and was summarized by standard descriptive statistical methods. χ^2^ test was used to compare categorical variables, whereas continuous variables were compared using student’s *t*-test. We defined PFS as survival without relapse or progression of hematological disease; we censored patients without disease or progression at the time of last follow up. We defined OS and NRM as death from any cause, and without evidence of relapse, respectively. We used the Kaplan-Meier method to calculate the probabilities of DFS and OS; and the cumulative incidence functions were used to estimate incidence of GVHD, Infections, viral reactivations, RI and NRM. All analysis was performed using SPSS version 26.0 and R version 4.0.5.

## Results

### Patients and transplant characteristics

A total of 289 consecutive patients were included in the study. Seventy-three patients (25%) received ATLG-30 and 216 Patients (75%) received ATLG-60with a tendency to give lower doses in more recent years. The median age at transplant was 57 years (range, 18–71) and 50 years (range, 18–74) in the ATLG-30 and ATLG-60 (*p* = NS), respectively. All patients, donor and transplant characteristics are listed in Table 1.

**Table 1:** Patients transplant and donor characteristics.

ATLG Dose	30 mg/Kg	60 mg/Kg	*P*
	*N* (%)	*N* (%)	
Total patients	73 (100)	216 (100)	
Patient age median (range)	57 (18–71)	50 (18–74)	0.98
Disease			0.22
ALL	3 (4)	19 (9)	
AML	37 (51)	108 (50)	
CML	2 (3)	6 (3)	
HL	1 (1)	0 (0)	
NHL	16 (22)	24 (11)	
MDS	6 (8)	19 (9)	
MDS/MPN	1 (1)	1 (0)	
MM	5 (7)	32 (15)	
PMF	1 (1)	4 (2)	
Others	1 (1)	3 (1)	
ECOG			0.06
0	16 (25)	50 (29)	
1	37 (59)	112 (65)	
2	8 (13)	8 (5)	
3	2 (3)	1 (1)	
KI at SCT median (range)	80(40-100)	80 (40–100)	0.38
Donor age median (range)	26 (19–53)	32 (18–61)	**0.022**
Donor/recipient CMV serology			0.17
D−/R−	32 (44)	73 (34)	
D−/R+	8 (11)	28 (13)	
D+/R−	4 (5)	30 (14)	
D+/R+	29 (40)	85 (39)	
Donor-recipient sex			0.55
No mismatch			
M-M	42 (58)	108 (50)	
F-F	12 (16)	32 (15)	
Mismatch			
M-F	14 (19)	54 (25)	
F-M	5 (7)	22 (10)	
ABO incompatibility			0.26
Isogroup	24 (33)	78 (36)	
Minor	21 (29)	63 (29)	
Major	22 (31)	45 (21)	
Bidirectional	5 (7)	28 (13)	
Year of transplant median (range)	2015 (2005–2019)	2014 (2006–2018)	0.25
CD34 x 10^6^/kg median (range)	8 (4–14)	8 (3-15)	0.19
Conditioning regimen			0.8
Bu -Based	37 (51)	111 (51)	
TBI-Based	23 (32)	73 (34)	
TMI + Bu+Cy	13 (18)	32 (15)	
TBI	23 (32)	75 (35)	0.2
TBI dose			
<12 Gy	8 (35)	20 (9)	
12 Gy	15 (65)	55 (73)	
Immune suppression			**0.044**
CNI + MMF	62	204	
CNI + MTX	10 (14)	9 (4)	
Other	1 (1)	3 (1)	
Disease status at SCT			**0.001**
CR	33 (67)	137 (85)	
CR1	19 (66)	103 (80)	
CR2	8 (28)	20 (16)	
CR3	2 (7)	4 (3)	
PR	4 (8)	14 (9)	
PD	12 (24)	10 (6)	
Follow up days median (Range)	337 (32–4843)	667 (20–3961)	0.73

Bold values indicate statistical significance *p* < 0.05.

*NS* Statistically not significant *p* ≥ 0.05, *ALL* Acute Lymphocytic Leukemia, *AML* Acute Myeloid Leukemia, *CML* Chronic myeloid Leukemia, *HL* Hodgkin’s Lymphoma, *NHL* Non-Hodgkin’s Lymphoma, *MDS* Myelodysplastic Syndrome, *MDS/MPN* Myelodysplastic syndrome-Myeloproliferative Neoplasm overlap, *MM* Multiple Myeloma, *PMF* Primary Myelofibrosis, *KI* Karnofsky Index, *SCT* Stem cell transplantation, *D/R* Donor/Recipient, *M* Male, *F* Female, *Bu* Busulfan, *TBI* Total body irradiation, *TMI* Total Marrow irradiation, *Cy* Cyclophosphamid, *CNI* Calcineurin inhibitor, *MMF* Mycophenolate Mofetil, *MTX* Methotrexate, *CR* Complete Remission, *PR* Partial Remission, *PD* Progressive Disease.

### Transplant outcomes

All transplant outcomes are summarized in Table 2.

**Table 2:** Transplant outcomes summary.

ATLG Dose	30 mg/Kg	60 mg/Kg	*P*
	*N* (%)	*N* (%)	
Leukocytes engraftment median (range) days	11 (8–23)	12 (8–27)	**0.009**
Platelets engraftment median (range) days	14 (9–53)	16 (8–237)	**0.002**
OS at 3 years	55%	51%	0.16
DFS at 3 years	45%	54%	0.18
NRM at 1 year	14%	11%	0.89
aGVHD			
Grade I-IV	66%	63%	0.35
Grade II-IV	47%	37%	0.09
Grade III-IV	19%	14%	0.2
cGVHD at 3 years			
Mild-moderate-severe	32%	36%	0.47
Moderate-severe	14%	14%	0.88
Severe	5%	4%	0.64
Infections			
Cumulative incidence of infections	49 (67%)	161 (75%)	**0.002**
CMV reactivation until day 100	31 (43%)	98 (45%)	0.06
EBV reactivation until day 100	15 (21%)	88 (41%)	**0.049**
IRM at day 180	8%	10%	0.7

Bold values indicate statistical significance *p* < 0.05.

*OS* Overall survival, *DFS* Disease free survival, *NRM* Non relapse mortality, *aGVHD* acute Graft versus host disease, *cGVHD* chronic graft versus host disease, *IRM* Infection related mortality.

### Engraftment

**Fig. 1:**
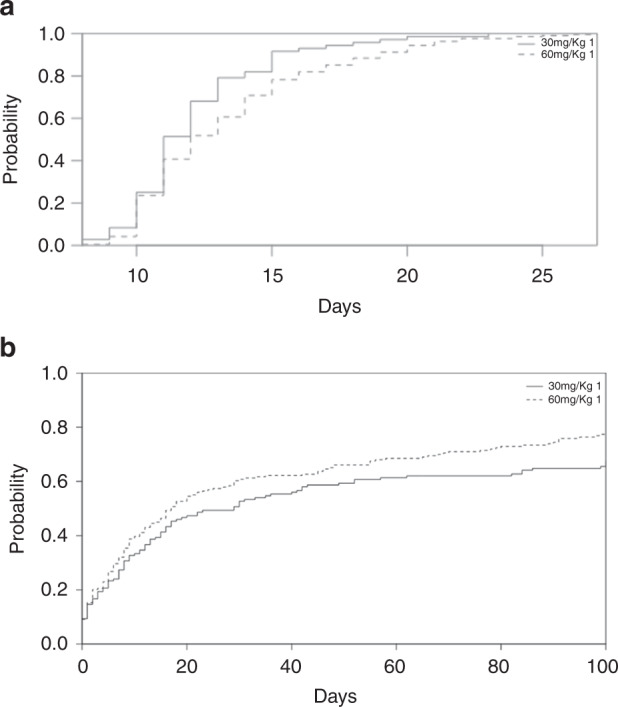
Comparison of two different ATLG dose for GvHD Prevention after unrelated allogeneic stem cell transplantation. **a** Comparison of neutrophil engraftment between 30 mg/kg and 60 mg/kg ATLG. **b** Incidence of infections after 30 mg/kg and 60 mg/kg ATLG.

Platelet and neutrophil engraftment were significantly delayed in ATLG-60 group when compared to the ATLG-30 group with a median of 11 days (range, 8–23) to neutrophil in the ATLG-30 vs 12 days (range, 8–27) in ATLG-60 group (*P* = 0.009) (Fig. 1a); and a median of 14 days (range, 9–53) to platelet engraftment in ATLG-30 group vs. 16 days (range, 8–237) in the ATLG-60 group (*p* = 0.011).

### Infections CMV and EBV reactivation

We observed no significant differences in incidence of CMV reactivation before day 100 (ATLG-30 43%, ATLG-60 45%). The overall incidence of infection before day 100 was significantly higher in the ATLG-60 (78%) when compared to the ATLG-30 (67%), *p* = 0.04 (Fig. 1b). The incidence of EBV reactivation before days 100 in the ATLG-30 group was lower than the ATLG-60 group (21% vs. 41% *p* = 0.049). IRM at 1-years was 10% in the ATLG-30 vs 11% in the ATLG-60 group (*p* = 0.7)

### Graft-versus-host disease

The cumulative incidence of aGVHD grade II-IV (47% vs 37%, *P* = 0.09) and III-IV (19% vs 14%, *P* = 0.2) were comparable in the ATLG-30 vs ATLG-60 groups, respectively. We observed a higher incidence of aGVHD grade IV in patients receiving ATLG-30 when compared to the ATLG-60 group (8% vs 0.5% *p* = 0.0002).

On univariate analysis, we observed no difference in the cumulative incidence of cGVHD all grade was (32% vs 37%, *p* = 0.47), moderate/severe (14% in both groups, *p* = 0.48) and grade severe (5% vs 4%, *p* = 0.64) were similar between the 30 mg/Kg ATLG and the 60 mg/Kg ATLG, respectively.

### Overall survival

**Fig. 2:**
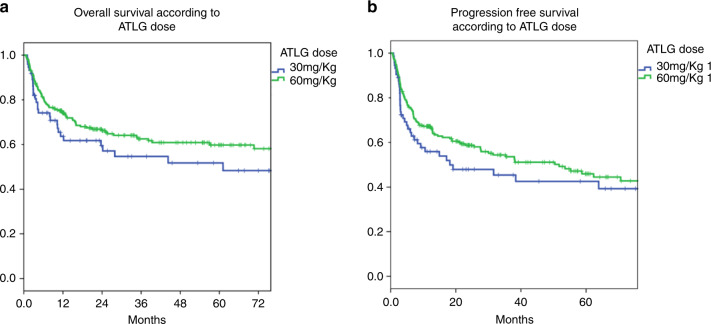
Comparison of two different ATLG dose for GvHD Prevention after unrelated allogeneic stem cell transplantation. **a** comparison of overall survival (OS) between 30 mg/kg and 60mg/kg ATLG. **b** comparison of disese-free survival (DFS between 30 mg/kg and 60 mg/kg ATLG and **c** comparison of cumulative incidence of non-relapse mortality (NRM) between 30 mg/kg and 60 mg/kg ATLG.

The estimated 3-year OS was 55% for patients in the ATLG-30 group and 51% in the ATLG-60 group (*p* = 0.16) (Fig. 2a). On univariate analyses patients’ age, patient and donor CMV serology, donor gender and disease status at transplant significantly affected OS. However, on multivariate analyses only Status at Transplant, Recipient CMV serology and Donor Gender were significant (Table 3).

**Table 3:** Multivariate analysis.

Multivariate analysis	OS HR [95%] CI *P*-value	DFS HR [95%] CI *P*-value
Patient age: (per 1 year increase)	1.52 (0.96–2.42) 0.08	1.33 (0.89–2) 0.17
ATLG Dose: 60 mg/Kg (ref) vs 30 mg/Kg	1.46 (0.85–2.53) 0.17	1.25 (0.77–2.03) 0.37
SCTchronology: 2 or more (ref) vs 1st	0.63 (0.37–1.07) 0.09	0.53 (0.34–0.85) 0.01
Status at transplant: not CR (ref) vs CR	0.52 (0.3–0.91) 0.02	0.55 (0.33–0.9) 0.02
Recipient CMV serology: Pos (ref) vs neg	0.47 (0.29–0.76) 0.002	0.54 (0.36–0.81) 0.003
DonorSex: Male (ref) vs Female	2.25 (1.38–3.67) 0.001	1.66 (1.05–2.62) 0.03

*HR* Hazard ratio, *CI* Confidence interval, *OS* Overall survival, *DFS* Disease free survival.

### Progression free survival

The estimated 3-year PFS was 45% for patients in the ATLG-30 group and 54% in the ATLG-60 group (*p* = 0.18) (Fig. 2b). On univariate analyses, older patients, negative recipient CMV serology, female donor, SCT chronology>1 and active disease at time of transplant were associated with decreased DFS. All the variables except patient age retained their negative impact on DFS in the multivariate analysis (Table 3).

### Non-relapse mortality

**Fig. 3:**
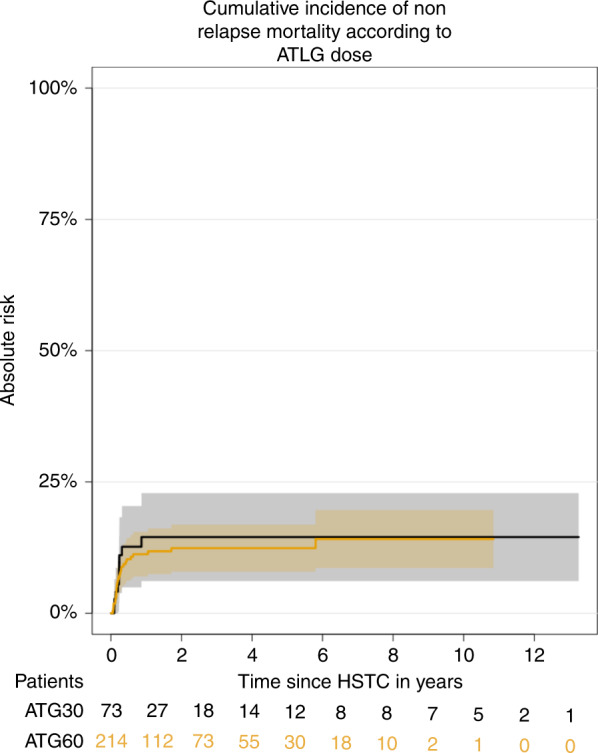
Comparison of two different ATLG dose for GvHD Prevention after unrelated allogeneic stem cell transplantation. Comparison of imnune reconstitution between 30 mg/kg and 60 mg/kg ATLG after transplantation regarding (**a**) gamma-delta T cells (**b**) naive T cells **c** Natural Killer (NK) cells and **d** naive B-cells.

The 2-years cumulative incidence of NRM was comparable between the two groups, with 14% vs 12% in the ATLG-30 and ATLG-60 (*p* = 0.89), respectively (Fig. 3). On Univariate analysis: older patients, female donor, and negative recipient CMV serology negatively affected NRM. These variables remained significant on multivariate analyses (Table 4).

**Table 4:** Multivariate analyses non relapse mortality.

NRM multivariate	HR [95%CI] *p*-value
Age (per 1 year increase)	**1.042 [1.02–1.07] 0.0015**
ATLG Dose: 30 mg/Kg (ref) vs 60 mg/Kg	0.93 [0.45–1.92] 0.84
DonorSex: Female (ref) vs Male	**0.33 [0.18–0.63] 0.0076**
Recipient CMV Serology: neg (ref) vs pos	**2.044 [1.002–4.17] 0.049**

Bold values indicate statistical significance *p* < 0.05.

*HR* Hazard ratio, *NRM* Non relpase mortality, *CI* Confidence interval.

### Immune reconstitution

**Fig. 4:**
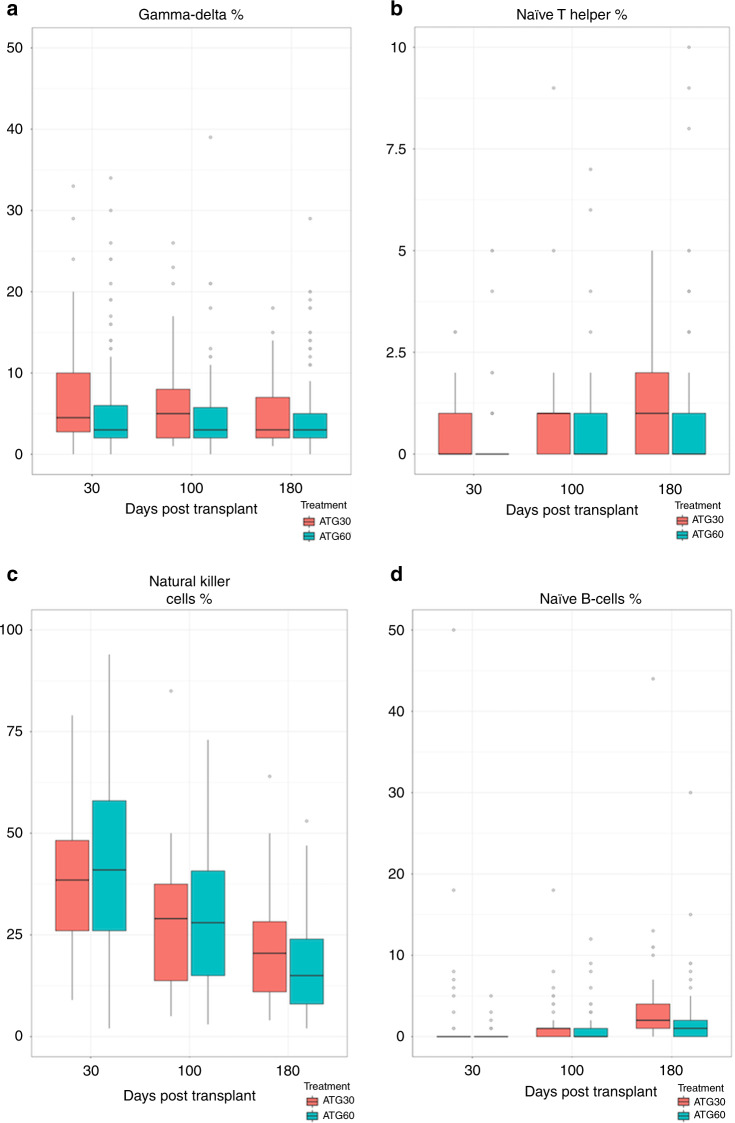
Comparison of Immune reconstitution. **a** of gamma-delta T cells post allo-SCT between 30 mg/Kg ATLG vs 60 mg/Kg ATLG. **b** of naïve T cells post allo-SCT between 30 mg/Kg ATLG vs 60 mg/Kg ATLG. **c** of NK-Cells post allo-SCT between 30 mg/Kg ATLG vs 60 mg/Kg ATLG. **d** of naïve B-cells post allo-SCT between 30 mg/Kg ATLG vs 60 mg/Kg ATLG.

At day +30, we observed a faster γδTcells reconstitution in the ATLG-30 group (*p* = 0.045) (Fig. 4a), however the values at day +100 and +180 were comparable between the two groups. Furthermore, helper naïve T-cells (CD4 + /CD45RA + ) (Fig. 4b) and NK cells reconstitution was faster at day +180 in the ATLG-30 group (Fig. 4c) and reconstitution of naïve B-cells (CD19+/CD27−/CD10+) was faster at days +30 and +180 in the ATLG-30 group (*p* < 0.0001) (Fig. 2d). All our data is summarized in Supplementary Table 1.

## Discussion

Although ATLG is recommended for GVHD prevention in allo-SCT [12], data on optimal ATLG dosing in the setting of MUD-PBSC is still lacking. Recently a consensus-based recommendation by an international expert panel recommended the use of 30 mg/Kg and 60 mg/Kg of ATLG for sibling and unrelated MAC allo-SCT, respectively [13].

A retrospective study conducted in 2003 compared two doses of ATLG (<60 mg/Kg vs 60 mg/Kg) in CML Patients undergoing MUD allo-SCT reports improved OS and DFS in patients receiving >60 mg/Kg ATLG. These differences were attributed to the higher incidence of severe aGVHD in the lower ATLG dose group. A number of 31 patients received a lower ATLG Dose with 58% receiving 20 mg/Kg ATLG and 36% receiving 40 mg/Kg ATLG [14]. Our findings fall in line with the study, as we reported a higher incidence of severe aGVHD on univariate and multivariate analysis in the lower ATLG dosing, however this did not impact any of the other outcomes. The discrepancy can be explained by the lower ATLG Doses in this study and improved supportive care in our population.

A study published from our center compared 30 mg/Kg ATLG with 60 mg/Kg ATLG in the MUD-allo-SCT setting between 1997–2005. ATLG was administered at a dose of 30 mg/kg on day –1 in the 30 mg/Kg group or 20 mg/kg/day on days –3 to –1 in the 60 mg/Kg group. This study reported a higher NRM in the ATLG-60 group, mainly due to increased incidence of infections in this group with no differences in incidences of aGVHD, cGVHD and Relapse [8]. Our results fall in line with the present study. We observed a higher incidence of infections in the ATLG-60 group, however, due to improved supportive care this had no impact on NRM.

In a multicenter, randomized, open label phase 3 Trial Locatelli et al. assessed the impact of two doses of ATLG (15 mg/Kg vs 30 mg/Kg over 3 days, from day −4 to −2) in children undergoing MUD-allo-SCT with MAC. They reported lower NRM, higher event free survival and OS in the lower ATLG dose group. In addition, higher doses of ATLG were associated with increased EBV and Adenovirus reactivation and increased IRM. They observed no significant differences in incidences of aGVHD and cGVHD between the two groups. Our results fall in line with Locatelli et al. In our we observed a higher incidence of EBV reactivation in the lower ATLG dose group, however, no differences in any of the long-term outcomes [15].

Butera et al. retrospectively evaluated the impact of two different anti-thymocyte globulin dose (Thymoglobulin, 5 mg/Kg vs 6.5 mg/Kg) in adults undergoing MUD-allo-SCT at three Italian centers [16]. They report an increased Infection related mortality in the higher ATG dose group, and no significant differences in any of the other outcomes. In our study, we observed a higher incidence of Infection, however no differences in Infection related mortality between the two groups. The differences in our outcomes can be explained in terms of the discrepancies in transplant characteristics. Butera et al. included both MAC and RIC, Bone Marrow and PBSC grafts and they reported a higher proportion of patients with 1HLA mismatch in the higher ATG dose group; while in our population Contrary in the present study, patients were more homogeneous, they all received MAC, PBSC with 10/10 HLA matched grafts. In addition, in contrast to ATLG Thymoglobulin may contain not only antibodies specific against mature T cell antigens but also antigens on the thymus-specific cells, thus directly impairing Thymic T cell regeneration and deepening the immunosuppression [17], thus explaining the increased IRM in patients receiving ATG in contrast to ATLG.

Gooptu et al. investigated the effect of ATLG on IR in MUD-PBSCT (*n* = 44 ATLG, *n* = 47 placebo) [18]. They reported improved CD3+, CD4+, CD8+, and Treg Lymphocytes in the Placebo group. They additionally reported delayed CD3+ and CD4+ Lymphocytes reconstitution up to 6 months. Our study falls in line with these findings, the CD3+ and CD4+ Lymphocytes did not normalize at last follow up.

Bacigalupo et al. compared in two randomized trials two different doses of thymoglobuline (15 mg/kg vs 7.5 mg/kg) in patients undergoing MUD-allo-SCT with MAC conditioning and BM grafts [19]. They reported decreased aGVHD in patients receiving higher doses (37% vs 69%). Two additional studies have compared different ATLG dosing, in haplo-identical and cord-blood setting [20, 21], both studies have reported increased infectious complications. Taking into consideration the basic differences in transplant types, our results fall in line with these findings, higher doses of ATLG have reduced incidence of grade IV aGVHD however at the cost of increasing infections in our patient’s population, without any impact on the long-term outcomes.

It has been recently suggested that ATG/ATLG dose should be calculated not only based on Body weight but also according to absolute Lymphocyte count on the first day of infusion [22, 23]. In two studies this approach has maximized the benefit by decreasing GVHD and reduced the risks of increased infections and relapse. This was also validated in a post hoc analysis of a RCT where lower ALC counts on first ATLG infusion day was associated with lower PFS and OS [24]. In our study we have not evaluated the impact of ALC on any of the outcomes.

γδTcells and NK-cells protect against viral and bacterial infections [25–28]. In addition administration of ATLG is associated with a faster γδTcell recovery [29]. Moreover, a recent study on the impact of γδTcells recovery on transplant outcomes in pediatric patients with acute leukemia reported improved DFS and OS in Patients with higher γδTcells [30], which was also confirmed in the adult populations [29, 31, 32], in addition they reported a decreased incidence of infections in patients with higher γδTcell count. The decreased γδTcells and longer period of aplasia in the ATLG-60 group can explain the higher incidence of infections in this group.

After allo-SCT the numbers of B cells normalize within a year [33–35]. In addition, ATLG induces apoptosis in CD20 + B cells and has shown to induce complement dependent cell lysis in myeloma cells [36, 37]. In our study the B cell have increased but did not normalize at day 180. In addition, the ATLG-60 group had a significantly lower naïve B-cell Population which supports the hypothesis that CD19 + B cells reduction by ATLG is dose dependent [38–41].

Early NK-cells recovery after allo-SCT has been previously reported and it has been postulated that ATLG spares NK cells when compared with other in vivo TCD strategies [42, 43]. In our study NK-cells recovered at day 30 in both groups and we observed no differences in cell count at all time points after transplant. However, we observed a higher percentage of NK cells in the ATLG 30 group. This can be clarified by the improved overall lymphopoiesis observed in the ATLG-30 group.

ATLG mediates in vivo T cell depletion by complement mediated cytolysis, Fas-receptor dependent apoptosis and antibody dependent cell mediated cytotoxicity [44]. In addition, Servais et al. analyzed the impact of ATG on IR post MAC PBSC allo-SCT [45]. It has been established that Tregs suppress GVHD without decreasing GVL [46], and that they accelerate T-cell IR in murine models [47]. Tregs in our study persisted after allo-SCT and we observed no significant differences in Tregs IR between both groups. This can be explained by previous reports of ATG selectively sparing Tregs [45].

Two studies have associated higher NKT-cell count with increased GVHD and relapse [48–50]. In our study, we observed early recovery of NKT cells without significant differences in IR between the two groups.

In both groups the CD8 + T cell compartment recovered at day +100, while the CD4 + Tcell compartment recovery was not achieved at day +180. This has been previously reported by Fehse et al. where CD8 + Tcells recovered within the first year after allo-SCT however CD4 + T cells failed to reconstitute within 2 years after allo-SCT [51]. This can be explained by the fact that: The reconstitution of the Tcell compartment after allo-SCT arises from both homeostatic peripheral expansion (HPE) of donor T-cells transferred with the graft and from novel production of naïve T-cells in the thymus [52, 53]. In patients receiving MAC most of the T-cells originate from HPE [54, 55]. In addition, HPE occurs more asymmetrically between T-cells, with CD8 + T-cells having higher proliferating potential by HPE when compared to CD4 + T-cells [45]. Moreover, It has been hypothesized that ATLG targets naïve T cells while sparing memory T cells [45]. Our study falls in line with previous findings, ATLG selectively compromised the recovery of naïve CD4^+^, memory CD4^+^, and naïve CD8^+^ cells, while it spared memory CD8 + T cells. However, in our study higher doses of ATLG had more pronounced effects on naïve T CD4 + Tcells, which supports the theory that ATLG exerts a dose dependent T cell effect [38, 56]. In addition, we observed a higher incidence of EBV reactivation in the ATLG-60 group. This can be attributed to the more pronounced severe depletion of naïve CD4 + Tcells and NK cells in this group.

More patients in the ATLG-30 group were not in CR at time of transplant, however we observed no differences in relapse incidence between the two groups which suggests a higher Graft versus Malignancy effect in the ATLG-30 group. However, it should be noted that more Patients in the ATLG-30 group had active disease at time of transplant, therefore calcineurin inhibitor may have differed between the two groups and may have influenced immune reconstitution.

Acknowledging the retrospective nature of our study, the choice of ATLG dose has significant impact on IR post MUD-allo-SCT, higher doses reduce aGVHD however they delayed engraftment, impair B-cell, γδTcells, NK and CD4 + T cell reconstitution and increase the risk for infection and EBV reactivation. However, this did not affect any of the long-term outcomes.

## Supplementary information

supplementary Table

## Data Availability

The datasets generated during and/or analysed during the current study are available from the corresponding author on reasonable request.
