# Supplementary material for: Impact of Anti-T-lymphocyte globulin dosing on GVHD and Immune reconstitution in matched unrelated myeloablative peripheral blood stem cell transplantation
Source: Bone Marrow Transplant. 2022 Jul 13;57(10):1548–55. doi: 10.1038/s41409-022-01666-x (PMC9532245; doi:10.1038/s41409-022-01666-x)
Supplement: Supplementary file 1 — supplementary Table [file 41409_2022_1666_MOESM1_ESM.pdf]

Cell type D30 MUD	Cell marker		30mg/Kg Median (range)	60mg/Kg Median (range)	P	Normal range
T-Lymphocytes	CD3+	(%)	51 (9-85)	36 (0.9-97)	**NS**	
		(cells /µl)	173 (15-1368)	101 (0-3438)	**NS**	**680-2000**
Activated T-Lymphocytes	CD3+/HLA-DR+	(%)	6 (0-44)	6.7 (0-69)	**NS**	
		(cells /µl)	15 (0-284)	17 (0-1971)	**NS**	**35-255**
Helper/Inducer T-Lym	CD3+/CD4+	(%)	7.25 (0-52)	5.79 (0-41)	**NS**	
		(cells /µl)	25 (0-340)	20 (0-544)	**NS**	**310-1185**
Supressor T/ Cytotoxic T-Lym	CD3+/CD8+	(%)	26 (5-65)	19 (0-90)	**NS**	
		(cells /µl)	107 (8-1107)	66 (0-3182)	**NS**	**180-820**
Helper/supressor T ratio	CD3+CD4+/CD3+CD8+		0.3 (0-3)	0.33 (0-2.23)	**NS**	
pan B-Lym	CD19+	(%)	0.65 (0-16)	0.8 (0-36)	**NS**	
		(cells /µl)	3 (0-27)	3 (0-445)	**NS**	**40-140**
B-subpopulation	CD19+/CD5+/CD1d+	(%)	0.7 (0-10)	0.65 (0-13.2)	**NS**	
		(cells /µl)	2 (0-45)	2 (0-116)	**NS**	**3-46**
	CD19+/CD27+	(%)	0.3 (0-1.7)	0.2 (0-7.8)	**NS**	
		(cells /µl)	1 (0-11)	1 (0-54)	**NS**	
Naïve B cells	CD19+/CD27-/CD10+	(%)	0 (0-50)	0 (0-5.3)	**0.003**	
		(cells /µl)	0 (0-116)	0 (0-26)	**<0.0001**	**760-2200**
NK cells	CD56+/CD3-	(%)	38 (9-79)	41 (2-94)	**NS**	
		(cells /µl)	125 (21-400)	124 (9-1280)	**NS**	**65-535**
NK T-cells	CD56+/CD3+	(%)	5 (1.3-20)	4.6 (0-69)	**NS**	
		(cells /µl)	19 (2-101)	15 (0-986)	**NS**	
Naïve CD4+ cells	CD4+/CD45RA+	(%)	0.2 (0-3)	0 (0-4.8)	**NS**	
		(cells /µl)	1 (0-8)	0 (0-47)	**NS**	**200-640**

Memory CD4+ cells	CD4+/CD45R0+	(%)	6.7 (0-50)	5.5 (0-36)	**NS**	
		(cells /µl)	26 (0-325)	16 (0-319)	**NS**	**145-530**
CD4+ Naïve/ memory ratio	CD4+/CD45RA+/CD4+/CD45R0+		0.03 (0-0.7)	0 (0-2.5)	**NS**	
Naïve CD8+ cells	CD8+/CD45RA+	(%)	8 (0-34)	6.7 (0-57)	**NS**	
		(cells /µl)	32 (0-436)	19 (0-1394)	**NS**	**290-570**
Memory CD8+ cells	CD8+/CD45R0+	(%)	10 (0.8-53)	6.8 (0-80)	**NS**	
		(cells /µl)	34 (1-605)	24 (0-1622)	**NS**	**25-320**
CD8+ Naïve/ memory ratio	CD8+/CD45RA+/CD8+/CD45R0+		1 (0-18)	0.9 (0-7.7)	**NS**	
Gamma-delta T cells	TCR gd /CD3+	(%)	4.45 (0-33)	3 (0-34)	**0.045**	
		(cells /µl)	14 (0-62)	10 (0-459)	**NS**	
T-reg cells	CD4+/CD25+/CD127+	(%)	2.4 (0-75)	2.25 (0-100)	**NS**	
Leukocytes		(cells x 10^3 /µl)	4.2 (1.6-145)	4.6 (1.2-59)	**NS**	
segmented neutrophils		(%)	62 (36-97)	67 (15-97)	**NS**	
		(cells x 10^3 /µl)	1.9 (0-11)	2.4 (0-45)	**NS**	
Lymphocytes		(%)	9 (1-30)	8 (1-64)	**NS**	
		(cells x 10^3 /µl)	0.34 (0.76-1.8)	0.32 (0.035-3.596)	**NS**	
Monocytes		(%)	19 (0-30)	12 (1-49)	**NS**	
		(cells x 10^3 /µl)	0.6 (0-1.3)	0.45 (0-2.5)	**NS**	

Cell type D100 MUD	Cell marker		30mg/Kg Median (range)	60mg/Kg Median (range)	P	Normal range
T-Lymphocytes	CD3+	(%)	57 (23-92)	56.3 (7.6-96.4)	**NS**	
		(cells /µl)	335 (21-3018)	346 (18-9956)	**NS**	**680-2000**
Activated T-Lymphocytes	CD3+/HLA-DR+	(%)	7.95 (0.8-41)	10.2 (1-73.3)	**NS**	
		(cells /µl)	56 (1-1372)	77 (2-7924)	**NS**	**35-255**
Helper/Inducer T-Lym	CD3+/CD4+	(%)	13.25 (2.5-35)	11.7 (0-42.9)	**NS**	
		(cells /µl)	88 (2-373)	87 (0-4637)	**NS**	**310-1185**
Supressor T/ Cytotoxic T-Lym	CD3+/CD8+	(%)	33.75 (13.5 - 74.4)	35.8 (0-85.3)	**NS**	
		(cells /µl)	184 (14-2515)	215 (0-4389)	**NS**	**180-820**
Helper/supressor T ratio	CD3+CD4+/CD3+CD8+		0.35 (0.11-1.3)	0.32 (0.02-2.4)	**NS**	
pan B-Lym	CD19+	(%)	4.5 (0-22.6)	4.15 (0-36.3)	**NS**	
		(cells /µl)	32 (0-220)	30 (0-449)	**NS**	**40-140**
B-subpopulation	CD19+/CD5+/CD1d+	(%)	0.6 (0-6.7)	0.7 (0-8.5)	**NS**	
		(cells /µl)	3 (0-60)	5 (0-113)	**NS**	**3-46**
	CD19+/CD27+	(%)	0.3 (0-1.5)	0.2 (0-2.4)	**NS**	
		(cells /µl)	2 (0-9)	2 (0-39)	**NS**	
Naïve B cells	CD19+/CD27-/CD10+	(%)	0.5 (0-17.6)	0.3 (0-11.7)	**NS**	
		(cells /µl)	3 (0-133)	2 (0-138)	**NS**	**760-2200**
NK cells	CD56+/CD3-	(%)	28.8 (5-84.57)	28 (2.8-72.55)	**NS**	
		(cells /µl)	149 (29-484)	177 (20-939)	**NS**	**65-535**
NK T-cells	CD56+/CD3+	(%)	4.85(0.4-17.7)	3.7 (0.22-67)	**NS**	
		(cells /µl)	33 (2-148)	25 (1-513)	**NS**	
Naïve CD4+ cells	CD4+/CD45RA+	(%)	0.5 (0-8.9)	0.2 (0-7.2)	**NS**	

		(cells /µl)	3 (0-44)	2 (0-110_	**NS**	**200-640**
Memory CD4+ cells	CD4+/CD45R0+	(%)	10.9 (3.7-32.3)	10 (1.9-30.6)	**NS**	
		(cells /µl)	76 (4-267)	72 (12-3308)	**NS**	**145-530**
CD4+ Naïve/ memory ratio	CD4+/CD45RA+/CD4+/CD45R0+		0.04 (0-0.82)	0.02 (0-0.38)	**NS**	
Naïve CD8+ cells	CD8+/CD45RA+	(%)	15.2 (2.7-52)	11.1 (0-70.9)	**NS**	
		(cells /µl)	89 (4-1741)	68 (0-1131)	**NS**	**290-570**
Memory CD8+ cells	CD8+/CD45R0+	(%)	15.75 (3.4-52.8)	18.9 (0-76.7)	**NS**	
		(cells /µl)	97 (10-976)	131 (0-3178)	**NS**	**25-320**
CD8+ Naïve/ memory ratio	CD8+/CD45RA+/CD8+/CD45R0+		0.95 (0.13-6.69)	0.69 (0-10.6)	**NS**	
Gamma-delta T cells	TCR gd /CD3+	(%)	4.65 (0.5-25.7)	2.97 (0-38.9)	**NS**	
		(cells /µl)	21 (1-215)	18 (0-670)	**NS**	
T-reg cells	CD4+/CD25+/CD127+	(%)	4.6 (0-23.7)	3.85 (0-37.5)	**NS**	
Leukocytes		(cells x 10^3 /µl)	4.2 (1.3-36)	4.3 (1.4-110)	**NS**	
segmented neutrophils		(%)	64 (42-81)	64 (2-90)	**NS**	
		(cells x 10^3 /µl)	2.4 (0-4.9)	2.7 (0-7.8)	**NS**	
Lymphocytes		(%)	15 (2-52)	18 (2-94)	**NS**	
		(cells x 10^3 /µl)	0.58 (0.07-3.38)	0.68 (0.15-10.8)	**NS**	
Monocytes		(%)	8 (2-17)	7 (1-21)	**NS**	
		(cells x 10^3 /µl)	0.27 (0-0.49)	0.28 (0-1.56)	**NS**	

Cell type D180 MUD	Cell marker		30mg/Kg Median (range)	60mg/Kg Median (range)	P	Normal range
T-Lymphocytes	CD3+	(%)	59.7 (20.8-88)	67.2 (16.5-94.27)	NS	
		(cells /μl)	448 (109-5108)	619 (8-3897)	NS	680-2000
Activated T-Lymphocytes	CD3+/HLA-DR+	(%)	9.9 (1.4-45.8)	12.65 (0-65.46)	NS	
		(cells /μl)	87 (8-1443)	139 (0-1406)	NS	35-255
Helper/Inducer T-Lym	CD3+/CD4+	(%)	14.43 (3.4-35.5)	14.4 (1.61-41.9)	NS	
		(cells /μl)	117 (14-919)	139 (0-652)	NS	310-1185
Supressor T/ Cytotoxic T-Lym	CD3+/CD8+	(%)	39 (12.5-74)	46.65 (0-89.37)	NS	
		(cells /μl)	316 (46-74)	391 (0-3634)	NS	180-820
Helper/supressor T ratio	CD3+CD4+/CD3+CD8+		0.4 (0.1-1.05)	0.32 (0.03-3.82)	NS	
pan B-Lym	CD19+	(%)	10.25 (0-32.7)	8.65 (0-63.8)	NS	
		(cells /μl)	109 (0-361)	84 (0-1213)	NS	40-140
B-subpopulation	CD19+/CD5+/CD1d+	(%)	1.1 (0-13.5)	0.8 (0-16.4)	NS	
		(cells /μl)	10 (0-806)	9 (0-468)	NS	3-46
	CD19+/CD27+	(%)	0.3 (0-1.4)	0.3 (0-4.7)	NS	
		(cells /μl)	3 (0-42)	3 (0-93)	NS	
Naïve B cells	CD19+/CD27-/CD10+	(%)	1.65 (0-44)	0.7 (0-30.2)	0.009	
		(cells /μl)	20 (0-320)	7 (0-225)	0.017	760-2200
NK cells	CD56+/CD3-	(%)	20.7 (4.4 -63.9)	14.8 (2-53)	0.035	
		(cells /μl)	171 (31-616)	151 (1-814)	NS	65-535
NK T-cells	CD56+/CD3+	(%)	3.55 (0-29.4)	2.9 (0.31-20.4)	NS	
		(cells /μl)	38 (0-1754)	30 (1-776	NS	
Naïve CD4+ cells	CD4+/CD45RA+	(%)	0.6 (0-20)	0.44 (0-10.4)	0.046	

		(cells /µl)	7 (0-185)	5 (0-147)	**NS**	**200-640**
Memory CD4+ cells	CD4+/CD45R0+	(%)	11.6 (2.5-23.9)	12 (0.2-49.5)	**NS**	
		(cells /µl)	99 (16-327)	125 (1-693)	**NS**	**145-530**
CD4+ Naïve/ memory ratio	CD4+/CD45RA+/CD4+/CD45R0+		0.06 (0-8)	0.04 (0-0.66)	**NS**	
Naïve CD8+ cells	CD8+/CD45RA+	(%)	14.55 (1.6-59.6)	15.9 (0-60)	**NS**	
		(cells /µl)	120 (9-1511)	144 (0-1970)	**NS**	**290-570**
Memory CD8+ cells	CD8+/CD45R0+	(%)	18.2 (1-64.3)	22.2 (0-63)	**NS**	
		(cells /µl)	140 (9-1399)	207 (0-2635)	**NS**	**25-320**
CD8+ Naïve/ memory ratio	CD8+/CD45RA+/CD8+/CD45R0+		0.83 (0.05-59.6)	0.69 (0-11.5)	**NS**	
Gamma-delta T cells	TCR gd /CD3+	(%)	3.31 (0.9-17.9)	2.6 (0-29.08)	**NS**	
		(cells /µl)	24 (3-477)	26 (0-741)	**NS**	
T-reg cells	CD4+/CD25+/CD127+	(%)	5 (0-14.8)	3.6 (0-41.7)	**NS**	
Leukocytes		(cells x 10^3 /µl)	5 (1.6-36)	4.8 (0.6-96)	**NS**	
Segmented neutrophils		(%)	60 (18-81)	60 (7-82)	**NS**	
		(cells x 10^3 /µl)	2.14 (0-6.3)	2.7 (0-6.3)	**NS**	
Lymphocytes		(%)	17 (3-65)	22 (1-78)	**NS**	
		(cells x 10^3 /µl)	0.77 (0.26-6)	1.03 (0.015-4.3)	**NS**	
Monocytes		(%)	9 (2-13)	8 (1-22)	**NS**	
		(cells x 10^3 /µl)	0.38 (0-0.77)	0.34 (0-1.092)	**NS**	
